# “I Stopped to Prove them Wrong:” Reasons for Discontinuing Daily Oral HIV Pre-exposure Prophylaxis Among Women at High Risk of HIV in Tanzania—A Convergent Mixed Methods Study

**DOI:** 10.1007/s10461-025-04879-5

**Published:** 2025-09-22

**Authors:** Wigilya P. Mikomangwa, Emmy Metta, Kåre Moen, Elia J. Mmbaga, Melkizedeck T. Leshabari, Stephen M. Kibusi, Christopher R. Sudfeld, Muhammad Bakari, Appolinary A. R. Kamuhabwa, Gideon Kwesigabo

**Affiliations:** 1 Department of Epidemiology and Biostatistics, Muhimbili University of Health and Allied Sciences, Dar es Salaam, Tanzania; 2 Department of Community Medicine and Global Health, University of Oslo, Oslo, Norway; 3 Department of Behavioural Sciences, Muhimbili University of Health and Allied Sciences, Dar es Salaam, Tanzania; 4 Department of Public Health, University of Dodoma, Dodoma, Tanzania; 5 Department of Global Health and Population, Harvard T.H. Chan School of Public Health, Boston, USA; 6 Department of Internal Medicine, Muhimbili University of Health and Allied Sciences, Dar es Salaam, Tanzania; 7 Department of Clinical Pharmacy and Pharmacology, Muhimbili University of Health and Allied Sciences, Dar es Salaam, Tanzania

**Keywords:** Pre-exposure prophylaxis, HIV, Female sex workers, Health belief model

## Abstract

High discontinuation of pre-exposure prophylaxis (PrEP) among high-risk women undermines its effectiveness. However, there is limited evidence on the reasons for PrEP discontinuation among female sex workers in sub-Saharan Africa. Exploring population-specific reasons for PrEP discontinuation will guide the design of client-centered strategies. Thus, we explored the reasons for PrEP discontinuation among female sex workers in Tanzania using mixed methods from 2022 to 2023 in Tanga, Tanzania. Participants were recruited through respondent-driven sampling and followed up for 12 months, and quantitatively interviewed at months 1, 6, and 12. In-depth interviews were carried out alongside the 12-month survey with purposely sampled female sex workers who had discontinued PrEP. Separate descriptive (and log-binomial regression) and thematic analyses were performed, and the insights were compared and integrated. We enrolled 313 participants with a median age of 27 years (IQR: 23–32). At 6 months, 61.5% (95% CI 54.3–68.4) had stopped taking PrEP for ≥ 3months, increasing to 67.4% (95% CI 60.2–74.0) at 12 months. Self-perceiving to be at “medium” to “high” risk of HIV had a 20% lower risk of stopping PrEP for ≥ 3months compared to self-perceiving to be at low or no risk (aRR 0.8, 95% CI 0.783–0.896). Participants discontinued PrEP because of medical and pharmacological challenges; perceived negative social norms and societal pressures; perceived undesirable pill characteristics and dosing schedules; low self-assessed HIV risk; perceived low benefit of PrEP; and low self-efficacy in adhering to PrEP. Our findings highlight the need for a multi-component intervention to promote PrEP retention.

## Introduction

HIV remains a global health challenge despite the availability of effective prevention strategies such as pre-exposure prophylaxis (PrEP). Globally, nearly 1.3 million people acquire HIV annually, of which 49.2% reside in the African region. Eastern and Southern Africa contribute about 34% of all new HIV infections globally [1]. Women aged 15 years and above contribute approximately 40% of all new HIV cases, whereas women at high risk of HIV, such as female sex workers, are 30 times more at risk of acquiring HIV than the general female population [1, 2]. The overall prevalence of HIV in Tanzania Mainland is 4.5% with women aged 45–49 years (13.0%) having a higher prevalence than men aged 50–54 years (8.4%) [3]. Although PrEP has been proven efficacious in clinical trials and demonstration studies, its full potential in reducing HIV incidence in programs is compromised by early discontinuation from PrEP [4]. About half of initiators worldwide discontinue PrEP use within 6 months post-initiation, with higher discontinuation rates in sub-Saharan Africa [4, 5]. Additionally, unsafe discontinuations (not using or using PrEP pills for less than 28 days after last HIV exposure) among daily PrEP users increase the risk of acquiring HIV, thus hindering the global effort to control the epidemic [4, 6, 7]. For instance, the HIV incidence among those who discontinue is estimated to be more than 3.4 per 100 person-years compared to about 2.53 per 100 person-years among non-PrEP users [8, 9]. Thus, continued HIV transmission among PrEP users hinders the efforts to realize the Joint United Nations Programme on HIV/AIDS (UNAIDS) goal of ending the AIDS epidemic as a public health threat by 2030 [10].

Various strategies have been studied to reduce rates of PrEP discontinuation by targeting one or more barriers. For example, mobile health (‘mHealth’) applications, mobile text message reminders and online counselling are promising in addressing premature discontinuation of PrEP by addressing communication barriers between PrEP users and healthcare workers, promoting a positive attitude towards PrEP and enhancing access to PrEP care [11, 12]. However, none of these implementation strategies has been adopted by the PrEP program in Tanzania. Also, community-based models have been adopted in addition to facility-based provision of PrEP services to improve uptake and retention [5]. Despite subsidizing and/or providing free PrEP services in most parts of the world, including in sub-Saharan Africa, the discontinuation rates are still high [13].

In sub-Saharan Africa, 43.3% of girls and women discontinue PrEP within 6 months of initiation [4]. Notably, female sex workers present with multiple discontinuation patterns characterized by temporary discontinuations of PrEP during periods of perceived low or no HIV risk [14]. High discontinuation rates are associated with mental health disorders, not having insurance and substance use, being female and young [5, 6, 13, 15]. The reasons for discontinuing PrEP among female sex workers reported in qualitative studies include experiencing side effects, perceiving oneself as being at low risk of acquiring HIV, feeling stigmatized, relying on condoms, having short PrEP pill refills, lacking family support, feeling burdened by the daily PrEP pill, experiencing an unfavorable PrEP clinic environment, facing high costs, and facing partner discouragement [4, 16, 17]. However, most of these reasons emanate from clinical trials and demonstration studies, with a paucity of information from real-world settings involving female sex workers [5]. Additionally, many existing studies do not report or interpret the findings in light of Health Behaviour Theories, thus reducing their implementation relevance in designing actionable strategies to address PrEP discontinuation [17, 18]. To date, no convergent model has been conducted in Tanzania to examine reasons for PrEP discontinuation among female sex workers.

While there has been an increase in the studies that explore reasons for PrEP discontinuation among members of the key population, with innovations of different PrEP dosing modalities to enhance adherence and retention in care [19], there is still a gap in our understanding of the reasons for PrEP discontinuation among female sex workers in Tanzania. Since the reasons for discontinuing PrEP could be population, setting or context-specific, we used a convergent mixed method to examine the reasons for PrEP discontinuation among female sex workers in Tanzania to inform and strengthen efforts for PrEP implementation. To provide a coherent explanation of the discontinuation from PrEP, we used the Health Belief Model (HBM) constructs to interpret and map the findings. The model was used to explore how female sex workers perceived HIV risk and the decision to discontinue PrEP use. We used the model constructs: perceived barrier, perceived susceptibility, self-efficacy and perceived benefit to explore reasons for discontinuing PrEP use.

## Methods

### Study Design

**Fig. 1:**
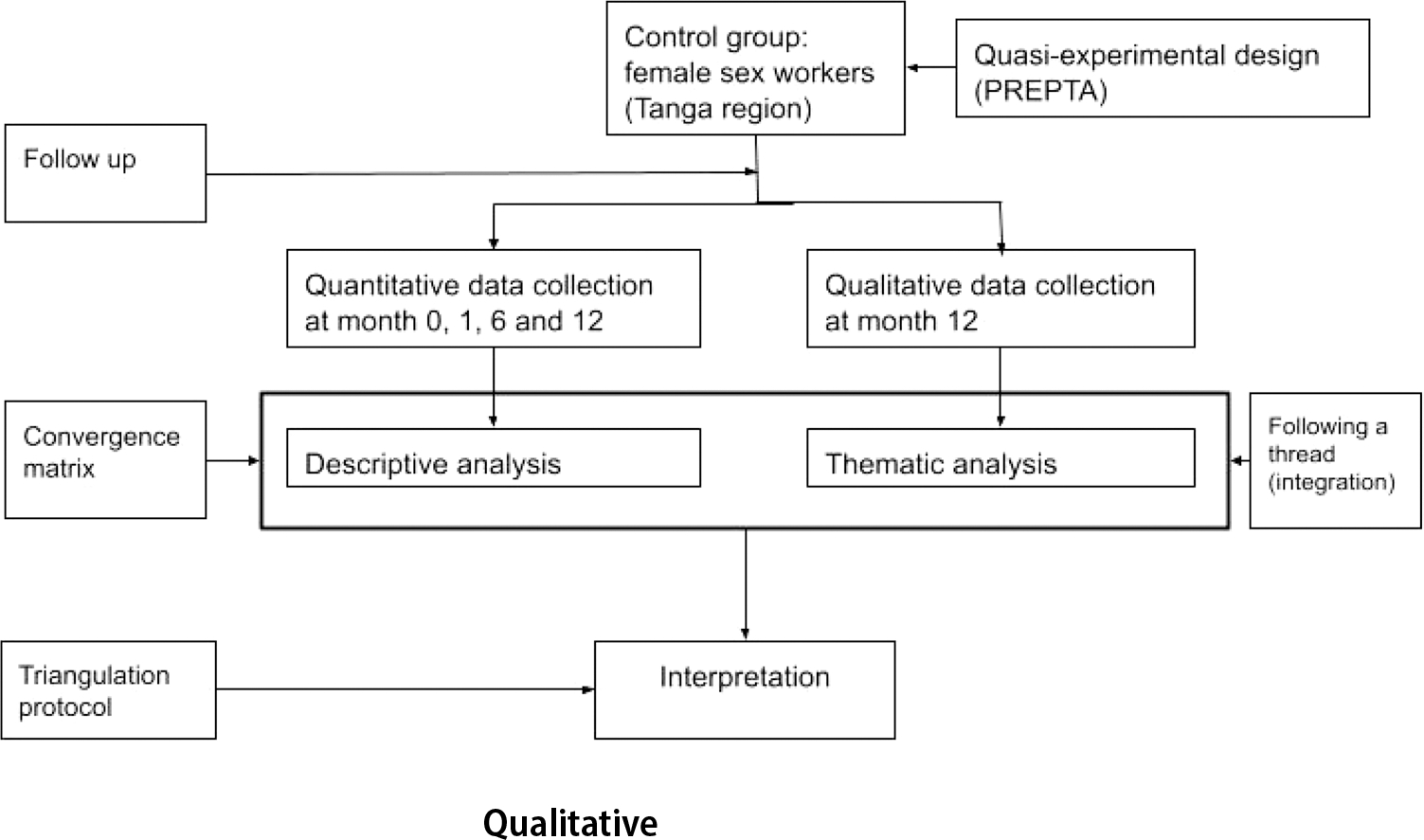
Study flow for convergence mixed method design among female sex workers in Tanga, Tanzania

We conducted a convergent mixed methods study involving a cohort of female sex workers recruited through respondent-driven sampling in the city of Tanga, Tanzania, each followed for 12 months in the period between February 2022 and June 2023, with data collection carried out at months 0, 1, 6 and 12. The quantitative data collection was part of the pragmatic quasi-experimental trial for HIV pre-exposure prophylaxis rollout in Tanzania (PREPTA). Details of the PREPTA methodology have been described elsewhere [20, 21]. At month 12, we also conducted a qualitative study using a phenomenological approach to explore the lived experiences and perspectives that led female sex workers to discontinue PrEP use. We used in-depth interviews (IDIs) with female sex workers who self-reported PrEP discontinuation to explore the reasons why they had stopped PrEP use. The quantitative and qualitative findings were analyzed separately and then triangulated to provide meta-themes that explain the reasons for PrEP discontinuation among female sex workers Fig. 1.

### Study Setting

The city of Tanga is located in the northeastern part of Tanzania along the coast of the Indian Ocean. It is the main commercial hub in the Tanga region and has the second-largest port in Tanzania [22]. The prevalence of HIV as of 2022/2023 among females aged 15–49 years was 3.5%, which was higher than among men (2.9%) [23]. In the Tanga region, 54.7% of female sex workers have reported having used at least a single dose of PrEP [24]. The study was conducted at Ngamiani Health Center, which is located in the center of the city.

### Data Collection

#### Quantitative

Electronic questionnaires in Swahili were used to collect quantitative data through interviews at months 0, 1, 6 and 12. We collected baseline characteristics of study participants at month 0, including sociodemographic characteristics, sexual behaviours, self-perceived risk of HIV, PrEP self-efficacy and social support. Data were collected longitudinally at months 1, 6 and 12 on PrEP use status, i.e., whether the participant was still using PrEP or had discontinued. In this case, the discontinuation was self-reported. Some of those who discontinued PrEP reported the reasons for discontinuing PrEP. During the interview, participants who had discontinued were asked to report when they discontinued PrEP after initiation. We also collected data on the reported side effects of PrEP. Moreover, changes in the pattern of sexual behaviour characteristics, such as condom use, and perceived risk of HIV, were collected during the follow-up period [20].

#### Qualitative

In-depth qualitative interviews (IDI) were conducted at month 12 follow-up among purposefully selected female sex workers who reported having discontinued PrEP. We aimed to get insight into the reasons that made them discontinue PrEP. Peer educators contacted female sex workers who had self-reported discontinuing PrEP for reasons other than HIV acquisition and renal insufficiency and scheduled interview dates. Before audio recording the interview (after consent), we documented (in the interview notes) the age, education level and self-reported period of using PrEP. The IDIs were conducted in Swahili (the national and official language in Tanzania) by the first author, guided by an interview guide. The guide had open-ended questions that aimed to capture what had caused women to discontinue PrEP. The interviews lasted for 15 to 25 min and were conducted in a quiet, dedicated room at the healthcare center for privacy and confidentiality, minimizing distraction, better audio recording and enabling participants to communicate freely. The interview was recorded using the Dictaphone application from Tjenester for sensitive data (“TSD”; Services for Sensitive Data), and the audio-recorded data were stored in the TSD database, which is hosted on a secure platform at the University of Oslo and requires multi-factor authentication for access. We conducted a total of 11 IDIs where the saturation of information was obtained within nine (9) IDIs and two (2) more interviews to confirm the saturation point i.e. confirmatory sampling.

#### Study Variables

Social demographic characteristics of study participants, including age, education level, living arrangements and having children. Self-efficacy was assessed using six items with five response options ranging from 0 (not at all confident) to 4 (very confident), and the Cronbach’s alpha was 0.84. We categorized PrEP self-efficacy into ‘low’ (score ≤ 18) and ‘high’ (score > 18). PrEP stigma was assessed using a 5-point Likert scale of 10 items (1 = Strongly disagree, 2 = Disagree, 3 = Neither disagree nor agree, 4 =Agree, 5 = Strongly agree), and Cronbach’s alpha was 0.88: We considered low PrEP stigma and high PrEP stigma if the scores were below 30 and higher than 30, respectively. Moreover, we adapted questions from the Duke-UNC Functional Social Support Questionnaire (FSSQ) to assess social support using a 5-Likert scale of 8 items, i.e., ranging from 1 = Much less than I would like to 5 = As much as I like (Cronbach’s alpha of 0.88), a score below 32 was considered inadequate social support [20].

#### Study Outcomes

The primary outcome was the reasons for discontinuing PrEP. The reasons for discontinuing PrEP use in the quantitative component were self-reported. Discontinuing PrEP use was either self-reported or documented (on the PrEP clinic file) stopping PrEP use. Secondary outcomes were the factors associated with stopping PrEP use for more than 3 months.

### Data Analysis

#### Quantitative

The quantitative and qualitative data analyses were conducted separately. We conducted a descriptive analysis of quantitative data and reported frequencies and percentages. The reasons for discontinuing PrEP which was assessed descriptively. We used weighted proportions for reasons for discontinuing PrEP that were reported repeatedly during the follow-up period; the denominator of each reason was used as the weight. Pearson’s chi-squared test and Fisher’s Exact test (where appropriate) was used to measure the association between categorical variables. The log-binomial regression analysis was used in determining factors associated with stopping PrEP use for more than 3months at p-value < 0.05 and 95% confidence interval (CI). The log-binomial regression model with robust standard error was used to obtain the relative risk (RR) since the proportion of stopping PrEP use was common (> 10%). Variables with P-values of < 0.2 during bivariate analysis were eligible for multivariable regression. The descriptive and regression analyses were conducted using STATA version 18.

#### Qualitative

The recorded IDIs were transcribed verbatim and checked for completeness. We manually analyzed the transcribed audio data. Thematic analysis was conducted following Braun and Clarke’s approach to reflexive thematic analysis [25]. The transcripts were read and re-read to become familiar with the content of the dataset. The lead author generated the initial codebook inductively (in English). Every piece of relevant data was coded during the initial coding. The initial codes were then reviewed by re-reading the transcripts. Codes capturing lived experiences, perspectives and opinions were used to identify the reasons for PrEP discontinuation. The codebook was reviewed and enriched by a second person, while a third person reviewed the generated findings. The coding and review were an iterative process. Codes with similar patterns and overlaps were grouped to create sub-themes. We then reviewed for potential themes by checking the theme against the refined codes: some codes were discarded if irrelevant to the study’s objective or reallocated to another theme. The themes were then refined and labelled deductively. The obtained themes were interpreted in the light of the Health Belief Model and supported by relevant quotes (translated from Swahili to English) related to discontinuation of PrEP use. The lead author drafted the initial report, and the co-authors reviewed it.

#### Convergence Model

The mixed methods approaches were fully integrated at the design, analysis, reporting and interpretation stages [26]. A triangulation protocol was used to converge (merge) the quantitative and qualitative findings [27]. The quantitative insights and qualitative themes were integrated using a convergence model to generate meta-themes that explain the circumstances for PrEP discontinuation among female sex workers i.e., cut across the findings from both quantitative and qualitative data. The emerging themes (qualitative) on the reasons for PrEP discontinuations were followed across the quantitative insights to develop meta-themes. We assessed whether the components of quantitative data converge (agree), expand, offer complementary information or dissonance with qualitative themes. The weaving narrative approach was used to report and interpret the overall results on a theme-by-theme basis by integrating the qualitative findings with the quantitative results [26] that confirm the reasons for PrEP discontinuation among female sex workers. For better visualization, we used a simplified joint display table that compares the quantitative results and qualitative findings as well as the convergence output.

## Results

### Baseline Characteristics of Study Participants

We enrolled a total of 313 female sex workers with a median age of 27 (IQR: 23–32) years. At baseline, most were unmarried (70.6%), lived with family (76.1%), perceived themselves to be at high risk of HIV (72.2%), and had not used a condom the last time they had sex with a client (63.3%). Almost all (99.6%) had high PrEP self-efficacy and 19.8% said that they experienced high levels of PrEP stigma. At month 6, most participants said they had stopped using PrEP more than 3 months ago (*p* < 0.001). Of these, 61.7% had children (*p* = 0.002). Also, 66.7% of participants who stopped PrEP for less than 3 months had travelled to other cities for sex work (*p* = 0.003) Table 1.

### Characteristics of Participants who Took Part in in-depth Interviews

The IDI involved 11 female sex workers with a median age of 27 (IQR: 23–33) years. The highest education level among participants was secondary school. All study participants reported having used PrEP for ≤ 3 months before deciding to discontinue Table 2.

### Trends and Attributes of PrEP Discontinuation

More than half of female sex workers stopped using PrEP in the previous 3 months or more. The proportion was 61.5% (95%CI: 54.3–68.4) at month 6 (*n* = 195) and increased to 67.4% (95%CI: 60.2–74.0) at month 12 (*n* = 187).

**Fig. 2:**
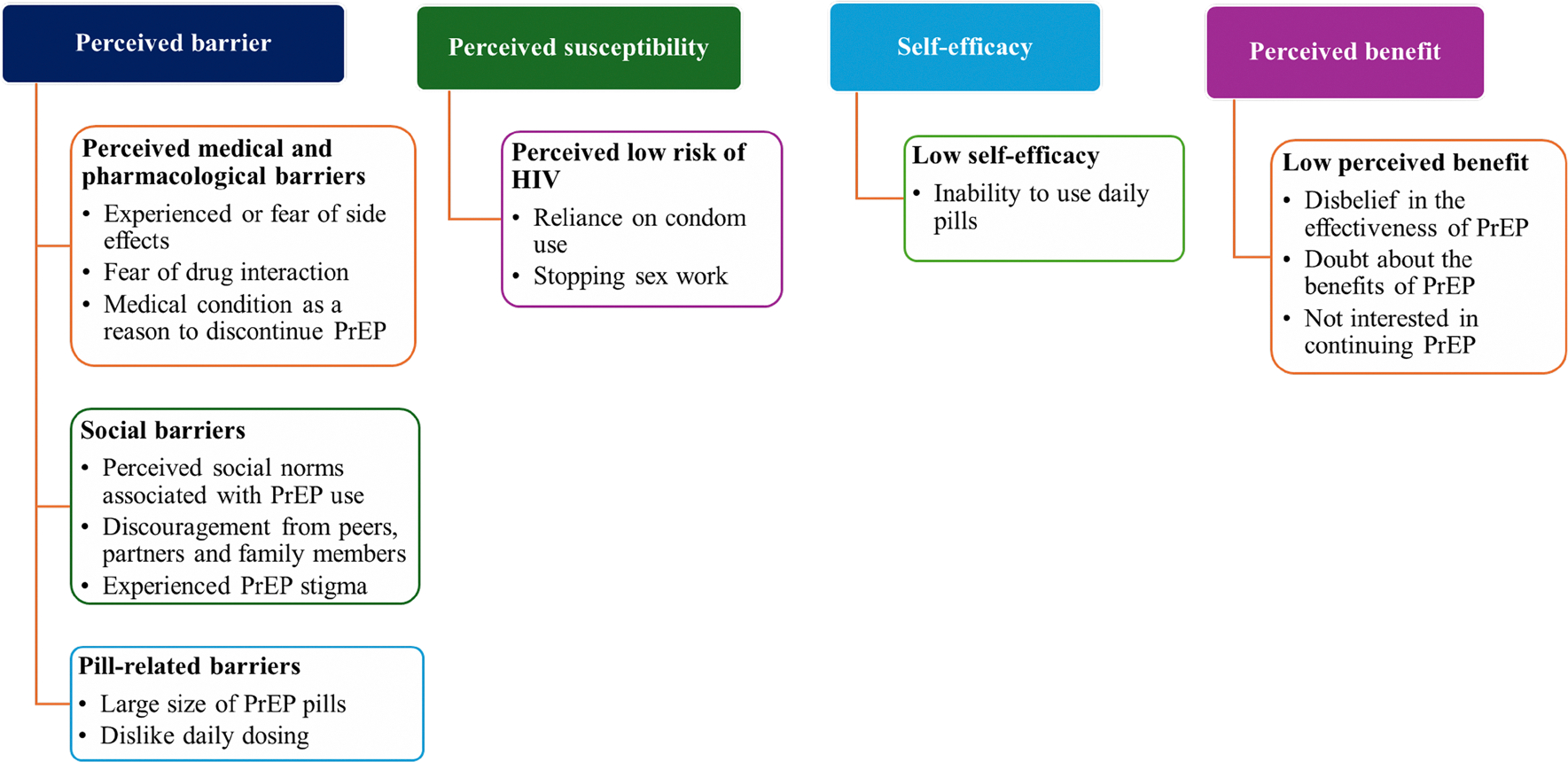
Distribution of themes and sub-themes on the reasons for discontinuing PrEP use by corresponding theoretical construct in the Health Belief Model

We then separately analyzed qualitative data. Six themes emerged on the reasons for PrEP discontinuation among female sex workers. The emerging themes were interpreted and mapped with the HBM constructs: Perceived medical and pharmacological barriers, social barriers, pill-related barriers, low perceived risk to HIV, low self-efficacy to PrEP and low perceived benefit of PrEP Fig. 2.

The qualitative themes convergence with all the quantitative results. Six meta-themes were developed on the reasons for PrEP discontinuation namely: The role of medical and pharmacological challenges in PrEP discontinuation; the influence of perceived social norms and societal pressure on PrEP discontinuation; pill characteristics and dosing schedule as drivers for discontinuing PrEP; the role of self-assessed HIV risk on PrEP discontinuation; low perceived benefit to PrEP as reason for PrEP discontinuation and; low self-efficacy influences PrEP discontinuation. Table 3.

### A. The Role of Medical and Pharmacological Challenges in PrEP Discontinuation

#### Experienced or Fear of Side Effects

**Fig. 3:**
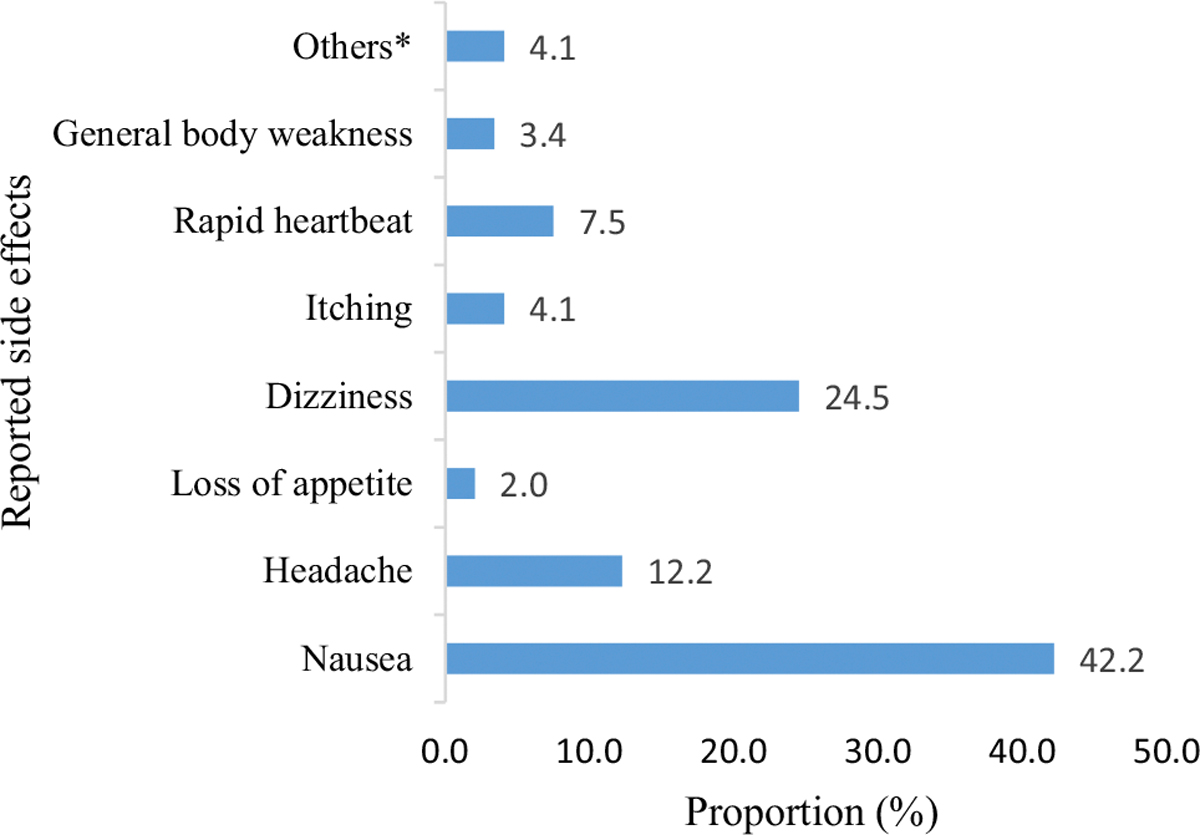
The proportion of self-report side effects following the use of PrEP pills among female sex workers at month 1 (*n* = 254)

Experienced side effects emerged as a barrier to PrEP continuation among female sex workers. At baseline, 98.7% of participants perceived that PrEP is not safe. Following the use of PrEP, 44.5% of female sex workers self-reported side effects. The most common side effects were nausea (42.2%), dizziness (24.5%) and headache (12.2%). However, 4.1% of female sex workers experienced other side effects such as vomiting, menorrhagia, dry throat, abdominal pain and polyphagia (Fig. 3). Of female sex workers who discontinued, 39.5% did so for reasons of self-reported side effects.

In the qualitative interviews, women reported similar side effects, such as headache, dizziness, nausea, vomiting, loss of appetite, irritation, body weakness, morning sickness-like symptoms, and heartburn. When explored further, some reported that the side effects had occurred in the first week of using the PrEP, while others stated that the side effects had lasted for more than two weeks. She said:

When I used these pills [PrEP] for the first time, I experienced a headache, dizziness or when I took them on an empty stomach, I became so weak. I also experienced menstrual bleeding, which was quite abnormal, though mild. I came here [health centre] and explained it to the clinician, but the bleeding continued, so I stopped using the pills. That’s it. *IDI 9*.

Some female sex workers had to cancel their working schedules as a result of the side effects. One participant stated:

The main problem was that I was worried about how I felt after taking the pills. Sometimes, when I had other things to attend to or go to work, I had to cancel because the pills made me feel so bad. *IDI 3*.

Other participants reported urinary tract infections (UTI) and irregular menstrual bleeding as side effects of PrEP. One IDI participant explained:

Every time I took the pills in the morning, I felt like vomiting and was weak. I thought that maybe I had malaria, I checked, and I had no malaria, no UTI. They asked if I was using contraceptive pills, and I told them I was not using contraceptives. After thinking, considering that I had no other medicines that I was using apart from these [PrEP pills], that’s why I decided to stop PrEP. IDI 6.

Some participants experienced side effects that may have been related to not following the recommended PrEP dosing schedule. For instance, one participant had experienced a rapid heart rate after taking PrEP every 12 h instead of the recommended 24-hour dosing interval. The participant explained:

I was experiencing a rapid heart rate; I never understood the reason for that situation. For almost five days consecutively I mixed the dosing schedules. If I took the pills at night, in the morning after waking up I took them again. That’s why maybe I experienced these adversaries until it was explained to me [by the healthcare provider] that I was supposed to take the pills, for example, if I chose at night, then every night. I then used several doses thereafter I recovered and stopped taking the pills [PrEP] altogether. IDI 3.

### Fear of Drug Interaction

Fear of drug interaction was a challenge for PrEP continuation. During the follow-up interviews, a small number of participants reported using other medications (4.2%) and discontinued PrEP due to fear of drug interactions. Some explained that they were afraid of mixing PrEP with other medications that had been prescribed to them, such as anti-diabetics. Moreover, some of the participants had conceived and were afraid of using PrEP while pregnant (4.5%). One participant said:

“Although I am still doing sex work, I had to stop PrEP, as I was afraid of what would happen if I mixed these anti-diabetics”. IDI 2.

### Medical Condition as a Reason to Discontinue PrEP

Being diagnosed with a medical condition was another hindering factor for PrEP use. For instance, one out of eight female sex workers (12.5%) said they had discontinued PrEP for various medical reasons. Probing in qualitative interviews found that some participants had been hospitalized and stopped using PrEP due to their poor well-being, including being diagnosed with an infection which needed medical attention or chronic illnesses such as diabetes. One interviewee echoed that due to her medical conditions she had to discontinue PrEP to have time to heal:

I was seriously sick; I was told had an infection though I don’t know what exactly it was. I was hospitalized for quite some time at the referral hospital. Although I had used the pills (PrEP) for like a month, I had to stop taking the pills even after discharge to give myself time to recover [...]. IDI 1.

### B. The Influence of Perceived Social Norms and Societal Pressure on PrEP Discontinuation

Perceived social norms emerged as an obstacle to continued PrEP use among female sex workers. They explained how community perceptions of PrEP led to discontinuation due to the visual similarity between PrEP and antiretroviral therapy (ART) packages (bottles) and pills. They highlighted that the community members, ranging from family level, friends and peers as well as partners or sexual clients, perceived the PrEP pills as ART (for treatment of HIV) due to their resemblance in shape, packaging and the need to take them daily. One participant explained how the family perceived PrEP use.

[...] I have a brother who, until now, believes that I am infected with HIV because of those pills. The pills look exactly like those for HIV. You see, those pills are a bit long in shape with a line in the middle, aren’t they? Even the ART pills look the same, their difference may be just colour and the bottle (packaging), but otherwise are the same. [...] When they see someone using these pills, they judge them as having HIV. So, I thought and decided to stop. *IDI 5*.

Participants expressed having experienced stigma from either family members, steady partners, sexual partners or peers due to the similarity of PrEP and ART, which led to the discontinuation of PrEP. At baseline, 84.6% expressed that the permanent partners would not support them taking PrEP. Due to social pressure, not having children was associated with a 30% increased risk of stopping PrEP use for more than 3 months compared to those with children (aRR, 1.3; 95%CI: 1.228–1.406) (Table 4). For them, using PrEP has affected their sex work as they are segregated by peers when on sex work; also, sexual clients get worried when they see them taking pills or see PrEP in their purses. They described that there is a spread of misinformation and rumours about being HIV positive. Participants further explained that they feared using the PrEP pills at home as they were afraid of stigma, fearful of being seen taking PrEP, and afraid of disclosing their PrEP use status due to fear of being stigmatized and perceived as HIV positive. She said:

I was afraid of being seen using those pills (PrEP), they never believed that I was using them for prevention, they thought that I was infected with HIV. [...] others were spreading rumours that I’m a brat and using ARVs, and people started pointing fingers at me. I felt like it was the right time to stop, so I had to stop to prove them wrong. IDI 8.

Participants further expressed that they sometimes felt forced to stop PrEP by their friends, sexual clients and family members as they would otherwise be labelled as HIV positive. Other female sex workers explained that steady partners were convinced that they had HIV since they used daily pills which looked exactly like ARVs. They described that the community members are used to seeing only HIV-infected people taking such kinds of pills. One participant narrated:

I had no privacy in taking the pills, I took them in front of my friends and family. I did not worry as I believed the pills were for my well-being, protecting me against HIV. But it reached a time, when I wanted to take, they discouraged me forcing me to stop. They spoke bad words about me, saying I am just the same as those [people living with HIV]. Friends, family were worried and said I should stop taking the pills if I am not infected. That’s why I stopped. IDI 4.

Many of the women felt that their peers, partners and family members pressured them to discontinue PrEP use. For example, at month 1, 21.6% indicated that they had received negative reactions after disclosing PrEP use to family members, partners and/or friends. During follow-up, 6.3% of those who stopped using PrEP reported that their partners or family members did not want them to use PrEP. Participants cited disapproval from their social circle as the reason for discontinuing PrEP use. Participants stated that they had no support from peers as they were the only ones using PrEP within the circle. One of the women narrated how her friends, had among their friends had thrown the PrEP pills in the trash bin a few days after initiating.

One of my friends took the pills [PrEP] from here [health facility]; she disposed of the pills the same day. We were sitting and chatting somewhere, she left the pack purposely, as some of our friends discouraged using PrEP. [...] others disposed of in trash bins as they do not want to be seen with the pills by their peers. IDI 1.

### C. Pill Characteristics and Dosing Schedule as Drivers for Discontinuing PrEP

#### Dislike of Pill Physical Characteristics and Daily PrEP Dosing

Pill physical characteristics and daily PrEP dosing emerged as bottlenecks for continuing PrEP use. In survey, 12.7% of female sex workers indicated that they disliked the daily dosing regimen and hence had decided to discontinue PrEP use. Moreover, 10.4% and 4.7%, respectively, did not like the taste and the colour of the pill. Also, 5.1% of participants were not satisfied with the PrEP medication and cited this as a reason to discontinue taking it. Probing further during IDI, we found similar explanations, including that some participants disliked PrEP pills’ taste and size and preferred other PrEP dosing regimens such as long-term injectable PrEP. As a result, they could not use PrEP for a long time, they decided to discontinue PrEP use. One participant stated:

I have a friend who never used those pills since we were given them at the health center. She just dislikes pills. Even some of us, just hate using pills. Better if it was injections, but not pills. I just don’t like pills. IDI 11.

#### The Large Size of PrEP Pills

PrEP pills were considered to be large, and their size acted as a hindrance to continued PrEP use among some women. In the survey, 20.1% of female sex workers complained about the PrEP pill being large, whereas 17.0% disliked the pill size and thus decided to discontinue PrEP. Similar findings were noted in the qualitative interviews, where participants demonstrated that the large size of the PrEP pills was among the reasons they decided to discontinue PrEP use. Participants expressed having experienced difficulties in swallowing the tablets. One participant stated:

The pill size is so large, at least if it were smaller, imagine when swallowing it gets stuck in the throat. At least, it should be made small, like half of the current size. Every time I looked at the pills I felt like collapsing. IDI 8.

### D. The Role of Self-assessed HIV Risk on PrEP Discontinuation

#### Reliance on Condom Use

Some female sex workers who discontinued PrEP self-perceived to be at low risk of HIV due to reliance on condom use. At baseline, 16.0% of all participants indicated that they perceived themselves to have low or no risk of HIV. In the follow-up period, 50.0% of those who discontinued PrEP self-perceived to be at low or no risk of HIV. Perceiving oneself to be at medium to high HIV risk was associated with a 20% reduced risk of stopping PrEP than perceiving oneself to be at low or no HIV risk (aRR 0.8, 95% CI: 0.783–0.896) (Table 4). During qualitative interviews, some participants explained that they were confident they were at low or no risk of HIV despite engaging in sex work due to their reliance on condoms as their prevention strategy. Despite acknowledging having been protected by PrEP in cases of condomless sex, some felt that condoms were enough to offer protection and that there was no need to take daily pills. For instance, one participant stated that:

For me, I don’t see any negative consequences of stopping (PrEP), because [...]I have never relied much on the pills, so I have never thought of any effect of stopping using them. I see there are so many other protections such as condoms, so even when I stopped PrEP, I normally use condoms. IDI 8.

In regression analysis, we noted that, those who perceived themselves to be at good (aRR; 0.8; 95%CI: 0.739–0.847) or very good health (aRR, 0.7; 95%CI: 0.677–0.775) had lower risk of stopping PrEP compared to those who perceived themselves to have poor health status. Table 4.

### Stopping Sex Work

Stopping sex work was linked to perceived low or no risk of HIV and was a reason to discontinue PrEP. Among participants who discontinued PrEP, 33.3% explained that they had done so because they had stopped sex work. In qualitative interviews, participants explained how they had discontinued PrEP since they were no longer at risk of acquiring HIV after stopping sex work. Some were no longer sex workers because they had married:

God has finally seen my friend, she got married. She decided to discontinue PrEP [...] fearing her husband if he realizes that she is using those pills, so she had to stop using the pills. IDI 3.

Other participants had discontinued PrEP after stopping sex work because they had gotten other jobs that paid better than sex work:

I discontinued PrEP since I stopped sex work, I got a job that pays well. That’s why. IDI 10.

### E. Role of Low Self-efficacy in PrEP Discontinuation

Female sex workers’ low sense of self-efficacy was among the reasons for discontinuing PrEP. At baseline, only 0.4% of female sex workers had low PrEP self-efficacy. Of participants who discontinued, 18.2% had a low sense of self-efficacy, which was expressed as a belief in the inability to take daily oral PrEP pills. Probing in qualitative interviews demonstrated a failure to use daily PrEP as a reason for discontinuing PrEP. When explored further, participants had coping mechanisms to overcome the challenges of taking daily pills. For instance, participants had to take PrEP pills with plenty of water, with food, after meals or even break the pill into two parts to address large pill size and discomfort. Despite these coping strategies, participants considered themselves incapable of continuing to take daily oral PrEP for a long time, considering that they were not sick and thus had to discontinue. One participant said:

[...] taking pills every day for what!. Just imagine taking pills every day while you are not sick. I felt it was too much for me, so I had to stop and rest. [...] I cannot take pills every day, for sure I cannot, daily pills, no! IDI 11.

Participants were of the opinion that they were tired of daily oral pills and that they did not want to be used to pills despite being sex workers, and thus, they just decided to stop. She said:

[...] In reality there are so many people who discontinue using these pills (PrEP) and the reasons are almost the same. [...] most of the reasons I hear points to being tired and inability to use pills daily [...]. IDI 9.

### F. Low Perceived Benefit to PrEP as the Reason for PrEP Discontinuation

Participants who discontinued PrEP reported having a low perceived benefit of PrEP as one of the reasons for discontinuing PrEP. The majority (86.3%) of female sex workers were worried of acquiring HIV despite using PrEP. IDI participants demonstrated disbelief and doubt about the benefits of daily PrEP. Participants reported a lack of trust in the benefits PrEP can offer in preventing HIV. They questioned the reason for taking the pills daily. Also, participants emphasized that they cannot directly trust PrEP. Some rated their trust to PrEP, by stating that they only trust PrEP by 50%. For example, one participant stated:

I cannot directly trust those pills (PrEP), you must use other protections like condoms even though we were told that it protects, but I cannot believe it directly. [...] If I have sex with an infected patient, I won’t acquire HIV, no I don’t trust it to that extent. [...] Although I somehow trust it for some percentage, like 50% but in reality, I don’t trust it that much. IDI 8.

## Discussion

This mixed-methods study examined the reasons for discontinuing PrEP among female sex workers in Tanzania. We found that female sex workers discontinued PrEP due to (a) various medical and pharmacological challenges associated with PrEP use; (b) discouraging social norms and societal pressure; (c) dislike of pill characteristics and dosing schedule; (d) self-perceived low own HIV risk; (e) perceived low benefit of PrEP; and (f) low PrEP self-efficacy.

Similar findings have been reported in a study based on in-depth interviews with female and male PrEP users in Kenya. They had discontinued PrEP due to experiencing side effects, PrEP stigma, and the large pill size [16]. A study involving female sex workers in South Africa reported difficulties in coping with the PrEP side effects and PrEP stigma as motivations to discontinue PrEP use [14, 28]. A study conducted in the USA reported that PrEP users discontinued PrEP because of side effects, contraindications or interactions [17] similar to our study in which female sex workers discontinued due to fear of contraindications and interactions, such as being pregnant, being diagnosed with diabetes and being hospitalized. Female sex workers’ perceived barriers to PrEP use act as obstacles and influence their decision to discontinue PrEP use.

When female sex workers discontinued PrEP use because of social norms and societal pressure, it was primarily linked to the common belief that people who use PrEP pills are HIV positive. The resemblance between pills used for prevention and treatment of HIV, including their packaging and daily dosing regimen, were highlighted as a common reason to discontinue PrEP because users were afraid of being labelled as HIV positive. People living with HIV are stigmatized in Tanzania [3] and female sex workers who discontinued PrEP could not tolerate being mislabelled as HIV positive. The misrecognition of PrEP users as HIV positive has been reported in Tanzania as barrier to PrEP use among adolescents and young girls [29]. The inability of community members to differentiate PrEP from ART for HIV treatment is due to a lack of community awareness of PrEP [18]. It has been noted that community members in Tanzania including potential PrEP users have low (less than 6.4%) awareness on the existence of PrEP [23, 30] highlighting the need to strengthen community sensitization on PrEP.

In addition, lack of family support and discouragement from partners was among the common reasons to discontinue PrEP. We noted that some female sex workers were disapproved of by people in their social circle for using PrEP, including by peers, friends and family members. Also, participants experienced negative reactions after disclosing PrEP use to family members, friends and peers as well as discouragement from partners. A previous qualitative study in Kenya also reported a lack of support from partners as a reason for stopping PrEP. In that study, participants expressed how unhappy their partners were when they took the PrEP pills [16]. Similarly, a study in Eswatini reported that female PrEP users discontinued PrEP due to disapproval from immediate family members [31]. Misunderstandings seem to play an important role here, and educational programs and awareness campaigns should be considered. It is equally important for family, friends, and community members to play an active role in motivating individuals at risk of HIV to continue using PrEP.

Perceiving oneself to be at low risk of HIV was another reason for discontinuing PrEP use. Also elsewhere has it been reported that perceived low HIV risk, or reliance on condom use, influences people to discontinue PrEP [14, 32–34]. A systematic review and meta-analysis of longitudinal studies reported a low risk of HIV as one of the common reasons PrEP users discontinue PrEP [4]. Female sex workers do not feel compelled to continue using PrEP if they perceive themselves as to be at a low or no risk of HIV, for example, if they stop sex work or use condoms [20]. This is in line with the guidelines on PrEP discontinuation which encourage PrEP users to safely discontinue PrEP during periods of low or no risk of HIV [7] hence preventing unnecessary drug exposure and side effects.

Self-efficacy is about an individual’s belief in their ability to continue using PrEP or overcome challenges associated with PrEP use [35]. Self-efficacy is crucial in ongoing PrEP use. Female sex workers in our study had low self-efficacy regarding PrEP use, and this was perceived to be among the reasons for discontinuing prophylaxis. Although only few studies have reported low self-efficacy as a reason for PrEP discontinuation, similar findings have been reported from some African settings where PrEP users describe their inability to use daily PrEP due to pill burden [16, 31]. A study in the USA reported bisexual men discontinued PrEP due to difficulty in adhering to taking PrEP daily [34]. Periodic assessment of PrEP self-efficacy is crucial in successful program implementation among members of the key population including female sex workers.

Some female sex workers who discontinued PrEP perceived PrEP to have few benefits. They expressed a lack of trust in PrEP as the reason for discontinuing PrEP. Similarly, a lack of trust in the effectiveness and relevance of PrEP has been reported by Unger and colleagues as a reason for discontinuation [17]. A qualitative study conducted in Tanzania involving adolescents and young girls reported disbelief in the effectiveness of PrEP in protecting them against HIV acquisition as a barrier to PrEP uptake [29]. Individuals’ disbelief toward PrEP represents the low perceived consequences of discontinuing PrEP use i.e., self-perceiving that, PrEP does not have any benefits to offer. For example, some female sex workers in our study expressed that, there are no adverse outcomes for discontinuing PrEP use. Counselling should be emphasized during routine PrEP use, and among female sex workers who discontinued PrEP while at substantial risk of HIV and/or considering restarting.

### Strengths and Limitations

The use of mixed design allowed for gaining a deeper and broader understanding of the reasons for PrEP discontinuation among female sex workers. The full convergence between the quantitative results and qualitative findings indicates validity, comprehensiveness and robustness of the generated evidence with a low risk of bias between the methods [36]. Aligning the findings with HBM underscores the areas that need to be addressed to optimize the retention in PrEP care among female sex workers. However, data were collected from female sex workers only at the single PrEP center, limiting its generalizability and transferability to other members of the key population and different parts of the country. Moreover, we did not assess the discontinuation practice as to whether female sex workers safely discontinued PrEP or not. Safely discontinuing PrEP is of paramount importance to avert HIV acquisition.

## Conclusion

Female sex workers discontinued PrEP due to a range of reasons: medical and pharmacological challenges associated with PrEP use; perceived social norms and pressure; unfavourable pill characteristics and PrEP dosing schedule; self-perceived low own HIV risk; perceived low benefits of PrEP use and low sense of PrEP self-efficacy. These findings indicate the multifaceted nature of circumstances that shape PrEP discontinuation among female sex workers. Interventions targeted at addressing these reasons will be useful in reducing the discontinuation rate of PrEP among female sex workers.

## Figures and Tables

**Table 1:** Distribution of baseline characteristics to reported time of discontinuing PrEP among female sex workers at follow-up month 6

Variable	Time (months) since stopped PrEP		
	
	≥ 3months, n (%)^∞^	< 3months, n (%)	p-values^~^

N	120 (61.5)	75 (38.5)	*0.000*
Age groups			
18–24	38 (31.9)	20 (26.7)	0.635
25–34	65 (54.6)	42 (56.0)	
35+	16 (13.4)	13 (17.3)	
Marital status			
Never married	89 (74.8)	48 (64.0)	0.108
Ever married	30 (25.2)	27 (36.0)	
Education level			
No formal education	11 (9.2)	7 (9.3)	0.799
Primary	58 (48.7)	33 (44.0)	
Secondary+	50 (42.0)	35 (46.7)	
Having children			
No	46 (38.3)	46 (61.3)	*0.002*
Yes	74 (61.7)	29 (38.7)	
Living arrangement			
Alone	11 (9.2)	8 (10.7)	0.612
Family	97 (81.5)	57 (76.0)	
Friends	11 (9.2)	10 (13.3)	
PrEP knowledge			
Low	72 (60.5)	51 (68.0)	0.291
High	47 (39.5)	24 (32.0)	
PrEP self-efficacy			
Low	0 (0.0)	1 (13)	0.209*
High	118 (100.0)	74 (98.7)	
Social support			
Inadequate	57 (50.4)	30 (42.3)	0.279
Adequate	56 (49.6)	41 (57.7)	
PrEP stigma			
Low	91 (77.1)	56 (76.7)	0.948
High	27 (22.9)	17 (23.3)	
Perceived HIV risk			
Medium/High risk	98 (82.4)	60 (80.0)	0.152
Low/No risk	20 (16.8)	11 (14.7)	
Don’t know the risk	1 (0.8)	4 (5.3)	
Perceived health status			
Very good	58 (49.6)	46 (61.3)	0.079
Good	44 (37.6)	26 (34.7)	
Fair/poor	15 (12.8)	3 (4.0)	
Condom use last time had sex			
Yes	65 (54.6)	40 (53.3)	0.861
No	54 (45.4)	35 (46.7)	
Travelled to other cities for sex work			
Yes	53 (44.5)	50 (66.7)	*0.003*
No	66 (55.5)	25 (33.3)	

*PrEP* HIV pre-exposure prophylaxis

~ Italicized p-values indicate significant association

* Fisher’s exact test was used to measure association

∞ Some variables do not total to 120 due to non-response

**Table 2:** In-depth interview participants’ characteristics

Participant number (IDI)	Age (years)	Education level	Time of PrEP use*

1	30	Primary	2 months
2	36	Primary	< 1 month
3	23	Primary	< 1 month
4	26	Primary	1 month
5	27	Primary	2 months
6	47	Primary	2 months
7	24	Primary	3 months
8	28	Secondary	3 months
9	27	Secondary	1 week
10	23	Secondary	3 weeks
11	20	Secondary	2 weeks

* Time of PrEP use was self-reported i.e., participants reported how long they used PrEP before discontinuing

**Table 3:** Quantitative and in-depth interview results on reasons for discontinuing PrEP with convergence output

Quantitative reasons for discontinuing PrEP	%	*N* *	Qualitative themes	Convergence output: meta-themes

			Perceived medical and pharmacological barriers	**Convergence**: The role of medical and pharmacological challenges in PrEP discontinuation
Side effects	39.5	19		
Medical reasons	12.5	16		
Use of many other medications	4.2	24		
Having pregnancy	4.5	22		
Experiencing PrEP stigma	4.5	22	Social barriers	**Convergence**: The influence of perceived social norms and societal pressure on PrEP discontinuation
Received negative reaction on PrEP use	21.6	245		
Partner or family do not want her use PrEP	6.3	16		
Disliked pill size	17	168	Pill related barriers	C**onvergence and expansion**: Pill characteristics and dosing schedule as drivers for discontinuing PrEP
Disliked taste of the pill	10.4	168		
Disliked pill colour	4.7	169		
Disliked daily PrEP dosing regimen	12.7	169		
Consistency use of condoms with clients	15.6	243	Perceived low risk of HIV	**Convergence**: The role of self-assessed HIV risk on PrEP discontinuation
Rejected condomless sex at increased payment	35	263		
Do not feel at risk of HIV	50	12		
No longer a sex worker	33.3	24		
No longer interested in daily PrEP	18.2	22	Low perceived benefit of PrEP	**Convergence**: Low perceived benefit to PrEP as reason for PrEP discontinuation
Difficulty in taking a pill daily	18.2	22	Low PrEP self-efficacy	**Convergence**: Low self-efficacy influences PrEP discontinuation

* Denominator used for determining the proportion of each observation (reason for discontinuing PrEP)

**Table 4:** Log-binomial regression analysis on the factors associated with stopping PrEP use for more than 3 months among female sex workers

Variables	cRR	95%CI	aRR	95%CI

Marital status				
Married	Ref		Ref	
Never married	1.2	0.937–1.627	1.1	0.835–1.463
Having children				
Yes	Ref		Ref	
No	1.4	1.120–1.692	1.3	*1.228–1.406*
Perceived HIV risk				
Low/No risk	Ref		Ref	
Medium/High risk	0.9	0.720–1.283	0.8	*0.783–0.896*
Don’t know the risk	0.3	0.052–1.833	0.4	0.068–1.874
Perceived health status				
Fair/poor	Ref		Ref	
Very good	0.7	0.511–0.876	0.7	*0.677–0.775*
Good	0.8	0.573–0.993	0.8	*0.739–0.847*
Travelled to other cities for sex work				
No	Ref		Ref	
Yes	0.7	0.566–0.890	0.7	*0.683–0.782*

*cRR* crude relative risk; *aRR* adjusted relative risk. Italicized indicates significant association (p-value < 0.05)

## Data Availability

All data are available upon reasonable request. The request for data can be sent to the PREPTA principal investigator: Prof. Elia J Mmbaga; elia.mmbaga@medisin.uio.no.
